# Prognostic impact of post-transplant diabetes mellitus in kidney allograft recipients: a meta-analysis

**DOI:** 10.1093/ndt/gfae185

**Published:** 2024-08-12

**Authors:** Mehmet Kanbay, Dimitrie Siriopol, Mustafa Guldan, Lasin Ozbek, Ahmet U Topcu, Ianis Siriopol, Katherine Tuttle

**Affiliations:** Division of Nephrology, Department of Medicine, Koc University School of Medicine, Istanbul, Turkey; Nephrology Department, “Sf. Ioan cel Nou” County Hospital, Suceava, Romania; “Stefan cel Mare” University, Suceava, Romania; Department of Medicine, Koc University School of Medicine, Istanbul, Turkey; Department of Medicine, Koc University School of Medicine, Istanbul, Turkey; Department of Medicine, Koc University School of Medicine, Istanbul, Turkey; Anaesthesia and Intensive Care Department, “Grigore T. Popa” University of Medicine and Pharmacy, Iaşi, Romania; “Grigore T. Popa” University of Medicine and Pharmacy, Iasi, Romania; Department of Medicine, Division of Nephrology, University of Washington, Seattle, WA, USA

**Keywords:** mortality, graft loss, kidney transplantation, new onset diabetes after transplantation, post-transplant diabetes mellitus

## Abstract

**Background:**

Post-transplant diabetes mellitus (PTDM) is a complex condition arising from various factors including immunosuppressive medications, insulin resistance, impaired insulin secretion and inflammatory processes. Its impact on patient and graft survival is a significant concern in kidney transplant recipients. PTDM's impact on kidney transplant recipients, including patient and graft survival and cardiovascular mortality, is a significant concern, given conflicting findings in previous studies. This meta-analysis was imperative not only to incorporate emerging evidence but also to delve into cause-specific mortality considerations. We aimed to comprehensively evaluate the association between PTDM and clinical outcomes, including all-cause and cardiovascular mortality, sepsis-related mortality, malignancy-related mortality and graft loss, in kidney transplant recipients.

**Methods:**

PubMed, Ovid/Medline, Web of Science, Scopus and Cochrane Library databases were screened and studies evaluating the effect of PTDM on all-cause mortality, cardiovascular mortality, sepsis-related mortality, malignancy-related mortality and overall graft loss in adult kidney transplant recipients were included.

**Results:**

Fifty-three studies, encompassing a total of 138 917 patients, evaluating the association between PTDM and clinical outcomes were included. Our analysis revealed a significant increase in all-cause mortality [risk ratio (RR) 1.70, 95% confidence interval (CI) 1.53 to 1.89, *P* < .001] and cardiovascular mortality (RR 1.86, 95% CI 1.36 to 2.54, *P* < .001) among individuals with PTDM. Moreover, PTDM was associated with a higher risk of sepsis-related mortality (RR 1.96, 95% CI 1.51 to 2.54, *P* < .001) but showed no significant association with malignancy-related mortality (RR 1.20, 95% CI 0.76 to 1.88). Additionally, PTDM was linked to an increased risk of overall graft failure (RR 1.33, 95% CI 1.16 to 1.54, *P* < .001).

**Conclusion:**

These findings underscore the importance of comprehensive management strategies and the need for research targeting PTDM to improve outcomes in kidney transplant recipients.

Key learning points **What was known:**

- Post-transplant diabetes mellitus (PTDM) is a common complication in kidney transplant recipients.
- The impact of PTDM on patient and graft survival, as well as cardiovascular mortality, has been recognized as a significant concern.
- Previous studies have produced conflicting findings regarding the effects of PTDM on various clinical outcomes.

**This study adds:**

- We demonstrated that PTDM is significantly associated with increased risks of all-cause mortality, cardiovascular mortality and sepsis-related mortality, and is also linked to a higher likelihood of overall graft failure.

**Potential impact:**

- This study underscores the need for enhanced management strategies for kidney transplant recipients to effectively address PTDM, highlights the critical importance of focused research to improve patient outcomes and reduce mortality and graft loss, and offers a clearer understanding of PTDM's specific risks to better inform clinical practices and interventions.

## INTRODUCTION

Post-transplant diabetes mellitus (PTDM) is multifaceted, stemming from underlying mechanisms such as insulin resistance, beta-cell dysfunction, and inflammatory processes [1–3]. Its incidence varies widely across studies, with reported rates ranging from 5% to 40% [4–7]. This variability is influenced by recipient characteristics, types of immunosuppressive treatments, and duration of follow-up post-transplantation [8–10]. PTDM typically occurs shortly after transplantation due to side effects of immunosuppressive drugs, genetic predisposition, age, and stressors of the transplant procedures and recovery [11, 12].

In kidney transplant recipients, PTDM can impact both short-term and long-term outcomes [13]. The prognosis of PTDM may affect mortality and graft survival [14, 15]. Evidence for PTDM risks is available from single-center and multicenter studies, albeit with varying degrees of certainty regarding prognosis. PTDM has been linked to increased mortality rates in several studies [15, 16], possibly due to its impact on overall morbidity and complications associated with diabetes [12]. Cardiovascular events, including major adverse cardiac events (MACE) and heart failure, and infections pose important mortality risks in this population [17–20]. However, its effect on graft survival is uncertain [15, 16]. Furthermore, it is noteworthy to mention that PTDM tends to manifest more frequently in transplant recipients of advanced age [21], thereby introducing complexity to direct observational comparisons between PTDM and control subjects.

A prior meta-analysis has provided valuable insights into the heightened risks associated with all-cause mortality and graft failure in PTDM [22]. However, a critical gap remains regarding cause-specific mortality, which we aim to address in this study to deepen our understanding of PTDM outcomes. By incorporating recently published studies featuring large cohorts, our objective is to reassess all-cause mortality and graft failure while also offering nuanced insights through a detailed breakdown of specific causes of mortality. Therefore, this systematic review and meta-analysis comprehensively evaluated the associations of PTDM with all-cause mortality, along with mortality due to specific causes including cardiovascular diseases, sepsis and malignancy, as well as graft loss. By comparing kidney transplant recipients who developed PTDM with those who were not diagnosed with PTDM, this study sought to provide insights that can inform clinical care and research needs to improve patient outcomes in the post-kidney transplant setting.

## MATERIALS AND METHODS

### Definitions

Both of the terms “NODAT” (New-Onset Diabetes After Transplant) and “PTDM” (Post-Transplant Diabetes Mellitus) were incorporated to ensure a thorough and comprehensive review of the literature. The terminology changes underscore the pathophysiological occurrence post-transplantation, emphasizing that these conditions are distinct from pretransplant diabetes mellitus based on their definitions. All relevant papers on these terms were included to encompass a broad range of perspectives and findings [23]. To maintain consistency in our article, we have opted to utilize the term “PTDM” throughout including the main text and the tables, even if the included sources vary in their use of terminology such as NODAT, or omit specific terminology altogether to describe individuals who develop diabetes mellitus following kidney transplantation.

Supplementary data, Table S3 provides comprehensive definitions of cardiovascular mortality, comprising all pertinent data concerning this particular cause. In essence, cardiovascular mortality encompasses myocardial infarction, cardiac arrest, sudden cardiac death and cerebrovascular events, facilitating the evaluation of cardiovascular-related fatalities.

### Search strategy and study selection

**Figure 1:**
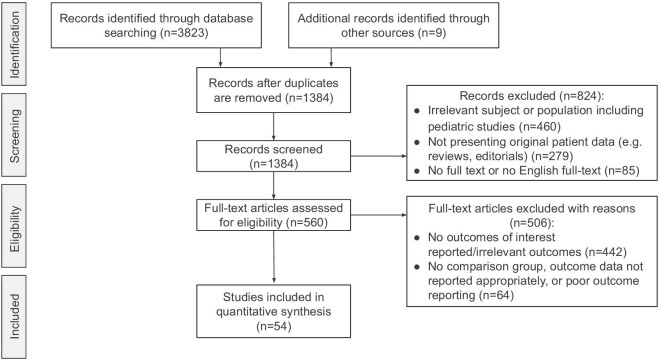
Flow diagram of the study selection process.

In this systematic review and meta-analysis, we adhered to the Preferred Reporting Items for Systematic Reviews and Meta-Analyses (PRISMA) guidelines for the design and reporting of the findings [24]. A thorough search strategy was designed with the assistance of a medical librarian to locate existing literature on mortality, cardiovascular diseases, infections, malignancies and graft outcomes among kidney transplant recipients with diabetes mellitus undergoing immunosuppressive therapy. We conducted a systematic search across multiple databases including PubMed, Scopus, Web of Science, Cochrane Library and Ovid/Medline, targeting studies published in peer-reviewed journals up to 3 August 2023. Our search terms encompassed “post-transplant diabetes mellitus,” “post-transplant diabetes,” “new-onset diabetes,” “kidney transplantation,” “renal transplantation,” “prognosis,” “mortality” and “graft survival.” Additional specifics regarding our search methodology and the keywords employed can be found in Supplementary data, Table S1. The articles were imported into the EndNote 21 and duplicates were checked and excluded automatically and subsequently re-checked manually by the authors (M.G., L.O. and A.U.T.). Three authors (M.G., L.O. and A.U.T.) independently screened the title and abstract of the identified articles for adherence to predetermined inclusion and exclusion criteria, ensuring a rigorous selection process. The selection of articles for the study was based on consensus among the authors, with any discrepancies discussed with a fourth author (M.K.). Subsequently, each full-text article found eligible in the screening process underwent a triple-blind review by the authors (M.G., L.O. and A.U.T.), supplemented by citation chaining to identify additional eligible articles. Any discrepancies regarding the eligibility of full-text articles were again discussed with the fourth author (M.K.). The study selection process is outlined in Fig. 1. The review protocol was not formally registered on any platform.

### Selection criteria

We systematically included studies reporting patients diagnosed with PTDM and comparing them with either patients with pretransplant diabetes or patients without diabetes before and after kidney transplant, provided they presented patient data and full-text availability, and reported outcomes such as rejection episodes, graft survival and cardiovascular mortality. Exclusion criteria comprised studies reporting patients with solid organ transplantation other than kidney, studies that do not report any post-transplant patients with diabetes, non-human studies, studies reporting only pediatric (age <18 years) patients and studies not reporting patient-level data, i.e. reviews, systematic reviews, meta-analyses, editorials, commentaries, book chapters, conference materials or those without a comparison group or full text. The outcomes of interest were all-cause mortality, cardiovascular mortality, sepsis mortality, malignancy-related mortality and graft loss. Studies not reporting these outcomes were excluded (Supplementary data, Table S1).

### Quality assessment

The assessment of study quality was conducted utilizing the Newcastle-Ottawa scale (NOS), which assigns a maximum total score of nine points, with a score of seven or higher indicative of high quality. Quality assessment using established criteria led to the exclusion of studies with poor grading, ensuring the robustness of the included literature (Supplementary data, Table S2).

### Data analysis

A random-effects model was used for this meta-analysis and expressed treatment effects as a risk ratio (RR) with 95% confidence intervals (CI) for the outcomes. Additionally, extracted hazard ratios (HR) from the study protocols were pooled using a random-effects model. Treatment effect was significant if *P* < .05. We assessed for heterogeneity in the treatment estimates using the Cochran Q test and the I^2^ statistic (with substantial heterogeneity defined as values >50%) [25]. Analyses were performed with the Review Manager (version 5.3, The Cochrane collaboration 2012).

## RESULTS

### Selection and description of studies

Our analysis included 53 studies, with one of them presented in two different manuscripts [26, 27]. The total number of patients included in our analysis was 138 917 (minimum 57 [28] and maximum 96 669 patients [29]). Although some studies included pretransplant diabetes [18, 26, 30–40], posttransplant prediabetes [41, 42], or transitory PTDM [43] alongside post-transplant normoglycemic and PTDM groups, we did not fully exclude them as they also involved our primary patient group of interest. However, those specific patient subsets with pretransplant diabetes, post-transplant diabetes or transient PTDM reported in these studies were not included in our analysis. The follow-up period was heterogenous with a minimum of 3 months [28, 44] and a maximum of 11 years median [45]; the follow-up period was not available in eight studies [16, 32, 34, 39, 46–49].

Ten studies were performed in North America (USA [16, 31, 37, 46, 50–55]), 16 in Europe (5 in the UK [32, 33, 40, 45, 56], 3 in Spain [26, 27, 42, 57], 2 in Portugal [48, 58] and Turkey [9, 59], and 1 in France [41], Italy [60], Slovakia [61] and Poland [44]), 5 in Africa (2 in Egypt [62, 63] and 1 in Tunisia [64], Sudan [65] and South Africa [66]), 18 in Asia (3 in China [29, 49, 67], Taiwan [35, 39, 68], India [30, 69, 70], Iran [34, 47, 71] and Korea [36, 43, 72], and 1 in Japan [73], Kuwait [74] and Bahrain [75]), 3 in Oceania (Australia [28, 38, 18]) and 1 in South America (Brazil [76]). Table 1 provides an overview of the baseline characteristics of the studies included in the meta-analysis.

**Table 1:** Baseline characteristics of included studies.

Author (year)	Study design	Population characteristics	Inclusion criteria	Exclusion criteria	Outcome measures
Abu Elmagd *et al*. [62] (2008)	Case–control	Total living-donor renal transplant recipients (*n* = 1580) ● Recipients developed diabetes after transplant (*n* = 286) ● Control group (*n* = 316)	● Living-donor renal allografts at Mansoura University between March 1976 and November 2004	N/A	● All-cause mortality at 8 and 15 years ● Graft survival
Ahmed *et al*. [65] (2017)	Prospective cohort	Total patients (*n* = 59) ● PTDM (*n* = 5) ● Non-PTDM (*n* = 54)	● Patients who underwent living-donor related renal transplant at the specified center between July 2012 and July 2013 ● Patients who were not diabetic before transplant	● Patients with diagnosed diabetic nephropathy as the cause of renal failure ● Patients who had diabetes before transplant	● All-cause mortality ● Graft survival
Al-Ghareeb *et al*. [75] (2012)	Retrospective cohort	Total adult kidney transplant recipients (*n* = 218) ● PTDM (*n* = 73) ● No-PTDM (*n* = 145)	● Adult patients who received a deceased-donor or a living-donor kidney transplant ● Followed-up at Al-Moayyed Nephrology and Transplant Unit, Salmaniya Medical Complex, Manama, Bahrain ● Transplantation occurred between January 1983 and December 2009	● Patients with graft failure or death within 1 month after transplant ● Graft loss caused by technical complications ● Patients with a diagnosis of DM before transplant (either as native kidney disease or comorbidity)	● All-cause mortality at 5 and 10 years ● Graft survival at 5 and 10 years
Alagbe *et al*. [66] (2017)	Retrospective cohort	Total number of patients meeting inclusion criteria (*n* = 111) ● Patients with PTDM (*n* = 20)	● Patients who underwent renal transplantation at Groote Schuur Hospital, Cape Town, South Africa, between 2004 and 2008 ● Patients without pre-transplant diabetes or primary non-function	● Patients who were lost to follow-up ● Patients with pre-transplant diabetes ● Patients with primary non-function of the transplanted kidney	● All-cause mortality ● Graft survival
Baron *et al*. [54] (2017)	Retrospective cohort	Total number of patients included (*n* = 279): Hispanic (*n* = 155) and Caucasian patients (*n* = 124) ● Hispanics with PTDM (*n* = 22) ● Hispanics without PTDM (*n* = 133) ● Caucasians with PTDM (*n* = 13) ● Number of Caucasians without PTDM (*n* = 111)	● Patients older than 18 years ● Patients who were not known as diabetic pre-transplant ● Patients who self-reported as Hispanic or Caucasian ● Patients who underwent first-time single kidney transplant ● Patients with a minimal follow-up of 12 months post-transplant	• Patients with ESRD secondary to DM ● Patients who did not undergo first-time single kidney transplant ● Patients with <12 months of follow-up post-transplant	● All-cause mortality ● Graft survival
Bzoma *et al*. [44] (2018)	Retrospective cohort	Total number of kidney transplant recipients included (*n* = 1424) ● Kidney transplant recipients with PTDM (*n* = 109) ● Patients with PTDM had their pairs of patients without PTDM included in the analysis (*n* = 74)	● Patients who underwent kidney transplantation at the Gdansk Transplantation Centre between 2001 and 2016 ● Patients with available data on PTDM ● Patients for whom paired kidney analysis was possible ● Patients with complete data for analysis	● Patients with incomplete data for analysis ● Patients with established DM before transplantation ● Patients who did not receive insulin therapy for at least 30 days after transplantation (for the diagnosis of PTDM) ● Patients who did not undergo paired kidney analysis ● Patients with missing or incomplete follow-up data	● All-cause mortality ● Graft survival
Cheng *et al*. [68] (2020)	Retrospective cohort	Total included patients (*n* = 425) ● Non-PTDM (*n* = 333) ● PTDM (*n* = 92)	● Adults (≥20 years old) ● Underwent renal transplantation between January 2000 and December 2018 ● Survived with a functioning allograft for ≥1 year after transplantation	● Patients with more than one organ transplant ● Post-transplant follow-up of <1 year ● Graft failure or death within the first year after transplantation ● Patients with pre-transplant diabetes	● All-cause mortality ● Cardiovascular mortality ● Sepsis mortality ● Malignancy mortality
Cheng *et al*. [49] (2022)	Prospective cohort	Total number of kidney transplant recipients included in the study (*n* = 495) ● Individuals with PTDM (*n* = 55) ● Individuals without PTDM (*n* = 440)	● First-time kidney transplant recipients ● Age range 18–70 years ● Complete clinical data available ● Patients followed up regularly for >1 year ● Absence of pre-operative DM ● Absence of history of hypoglycemic drug use ● Fasting blood glucose <7.0 mmol/L ● HbA1c <6.5% before kidney transplantation ● No combined organ transplantation ● No co-administration of azole antifungal agents	● Age <18 years ● History of DM ● History of hypoglycemic drug use ● Fasting blood glucose ≥7.0 mmol/L ● HbA1c ≥6.5% before kidney transplantation ● Combined organ transplantation ● Co-administration of azole antifungal agents ● Patients lost to follow-up	● Graft survival
Choi *et al*. [36] (2013)	Retrospective cohort	Total number of renal transplant recipients (*n* = 565) ● Non-PTDM group (*n* = 436) ● PTDM group (*n* = 129): pre-existing DM (*n* = 63) and PTDM (*n* = 66)	● Patients who received renal transplants at Hanyang University Transplantation Center between January 1990 and December 2011 ● Patients with available data on diabetes status and graft outcomes	● Patients with incomplete data on diabetes status or graft outcomes	● Graft survival at 5 and 10 years
Cosio *et al*. [31] (2002)	Retrospective cohort	Total adult renal allograft recipients: (*n* = 1811) ● No diabetes before and after transplant (no DM) (*n* = 1186) ● Diabetes before transplant (DM): (*n* = 332) ● PTDM (*n* = 293)	● Adult renal allograft recipients who received their first kidney transplants at The Ohio State University between 1983 and December 1997 ● Patients who maintained graft function for at least 6 months post-transplant	● Patients who did not maintain graft function for at least 6 months post-transplant ● Patients with missing data on key variables ● Patients treated with FK506 (Prograf) or sirolimus (rapamycin)	● All-cause mortality ● Cardiovascular mortality
Cotovio *et al*. [48] (2013)	Case–control	Kidney transplant recipients evaluated (*n* = 648) ● Recipients who developed PTDM (*n* = 83) (12.8%) ● Number of recipients included in the comparison: PTDM (*n* = 47) vs non-PTDM (*n* = 47)	● Kidney transplant recipients between 2005 and 2009 ● Diagnosis of PTDM according to the PTDM 2003 International Consensus Guidelines ● Recipients with a paired control who did not develop PTDM ● Minimum follow-up of 6 months	● Recipients whose pair also developed PTDM ● Recipients for whom only one kidney was available for transplantation	● All-cause mortality ● Cardiovascular mortality ● Sepsis mortality ● Graft survival
Dedinská *et al*. [61] (2015)	Retrospective cohort	Total number of patients (*n* = 167) ● Number of patients with PTDM (*n* = 64) ● Number of patients without PTDM (control group) (*n* = 103)	● Patients who underwent primary kidney transplantation from a deceased donor ● Patients who were monitored for at least 12 months after transplantation	N/A	● Cardiovascular mortality ● Graft survival
Demirci *et al*. [9] (2010)	Retrospective cohort	The study included kidney transplant recipients who underwent renal transplantation (*n* = 555) ● Post-transplant DM (*n* = 102) ● No PTDM group (*n* = 453)	● Patients who received kidney transplants at the center between 1989 and 2003 ● Patients with available data on the development of PTDM ● Patients with complete demographic and clinical information	● Patients with diabetic nephropathy as the primary kidney disease (*n* = 3) ● Patients who developed primary non-function of the transplanted kidney (*n* = 5) ● Patients with inadequate follow-up (*n* = 13)	● All-cause mortality at 5 and 10 years ● Graft survival ● DCGL
Dienemann *et al*. [37] (2016)	Retrospective cohort	● No diabetes (*n* = 980) ● Pretransplant diabetes (*n* = 563) ● PTDM (*n* = 447)	● Recipients who received their first kidney transplant at the specified center between 1 January 1996 and 31 December 2012 ● Aged >18 years ● Functioning transplant for at least 1 year ● Sequential and simultaneous multi-organ recipients	● Patients with <12 months of follow-up ● Patients with allograft loss, death, or those who lost-to follow-up within the first year posttransplant	● All-cause mortality ● Graft survival ● DCGL
Ducloux *et al*. [41] (2005)	Prospective cohort	Total consecutive stable renal transplant recipients (*n* = 357) ● Patients with PTDM (*n* = 39) ● Patients without PTDM (*n* = 318): 281 with normal fasting plasma glucose, 37 with borderline fasting plasma glucose	● Consecutive stable renal transplant recipients with a transplant duration of >12 months ● No episode of acute rejection ● Serum creatinine level <400 μmol/L ● No history of DM before transplantation	● History of DM before transplantation ● Acute rejection episodes ● Serum creatinine level of ≥400 μmol/L	
Einollahi *et al*. [34] (2008)	Case–control	Total number of kidney transplant patients included (*n* = 222) ● Patients with DM (*n* = 111) ○ Type 1 DM (*n* = 36) ○ Type 2 DM (*n* = 20) ○ PTDM (*n* = 55) ● Randomly selected kidney transplant recipients without DM (*n* = 111)	● Adult kidney transplant patients (age >20 years) ● Recipients of kidney allografts from living donors ● Followed up at the outpatient clinic in Baqiyatallah Hospital between 1986 and 2001	N/A	● All-cause mortality ● Graft survival
Fernández-Fresnedo *et al*. [57] (2003)	Retrospective cohort	Total number of renal transplant patients analyzed (*n* = 503) ● Patients with PTDM (*n* = 85) ● Patients without pretransplant DM (non-PTDM) (*n* = 418)	● Renal transplant patients between 1985 and 2000 ● Patients with functioning kidneys for more than 1 year ● Patients without pretransplant DM	• Pretransplant diabetic patients	● All-cause mortality
Gnatta *et al*. [76] (2010)	Prospective cohort	Total number of individuals (*n* = 413) ● Individuals with PTDM (*n* = 85) ● Individuals without PTDM (*n* = 328)	● Patients aged 18 years or older who received a primary or a second kidney transplant from a living or deceased donor ● Patients treated with tacrolimus (TAC), cyclosporine (CyA), or sirolimus (SIR) plus steroid therapy ● Minimum posttransplant follow-up of 6 months	N/A	● All-cause mortality ● Graft survival
González-Posada *et al*. [26] (2004)	Retrospective cohort	Total number of individuals (*n* = 3365 adult kidney allograft recipients) ● Individuals with pre-existing DM as an underlying disease (*n* = 156) ● Number of individuals with (PTDM) (*n* = 251) ● Number of individuals without diabetes (*n* = 2958)	● Adult kidney allograft recipients (>17 years) ● Recipients receiving a single kidney that was functioning at the end of the first year after transplantation	N/A	● DCGL
González-Posada *et al*. [27] (2006)	Retrospective cohort	Total number of individuals: 3365 adult kidney allograft recipients ● Individuals with PTDM (*n* = 251) ● Individuals with DM as the primary disease (*n* = 156) ● Individuals without diabetes (control or non-PTDM) (*n* = 2958)	● Adult kidney allograft recipients (>17 years) ● Recipients receiving a kidney that was functioning at the end of the first year after transplantation	● Graft loss during the first year after transplantation ● Other organ or double kidney transplants ● Incomplete data availability	● All-cause mortality
Hussain *et al*. [40] (2022)	Retrospective cohort	674 living-donor, 784 DBD, 243 DCD renal transplant recipients (*n* = 1757) ● No diabetes (*n* = 1307) ● PTDM (*n* = 243) ● Pre-existing diabetes (*n* = 207)	● All consecutive kidney-alone transplants performed at a single center in the UK between 1 January 2007 and 30 June 2018	● Aged <18 years ● Recipients of multiple organs	● All-cause mortality
Jeon *et al*. [72] (2023)	Retrospective cohort	293 living-donor, 278 deceased-donor renal transplant recipients (*n* = 571) ● PTDM (*n* = 153) ● No PTDM (*n* = 418)	● Kidney recipients who underwent transplantation from 1994 to 2017 and were followed until 2021 in a single center	● Diabetes before transplant ● Aged <18 years ● Lost to follow-up ● Two or more graft transplantations	● All-cause mortality ● Graft survival ● DCGL
John *et al*. [30] (2001)	Prospective cohort	Renal transplant recipients (*n* = 1414) ● No diabetes (*n* = 1138) ● PTDM (*n* = 174) ● Pre-existing diabetes (*n* = 102)	● Patients who received a renal transplant in a single center from 1986 to 1999	● Multiple-transplant subjects ● Patients who developed pre-transplant tuberculosis	● All-cause mortality
Johny *et al*. [74] (2002)	Retrospective cohort	502 living-donor, 50 deceased-donor renal transplant recipients (*n* = 552) ● No diabetes (*n* = 435) ● PTDM (*n* = 117)	● Renal transplant recipients followed up in a single transplant center from January 1983 to January 1998	● Pre-existing diabetes	● All-cause mortality ● Cardiovascular mortality ● Sepsis mortality ● Malignancy mortality ● Graft survival
Joss *et al*. [33] (2007)	Retrospective cohort	16% living-donor, 84% deceased-donor renal transplant recipients (*N* = 787) ● No diabetes (662) ● PTDM (*n* = 55) ● Pre-existing diabetes (*n* = 70)	● All patients who received a renal transplant between 1994 and 2004 in a single center	N/A	● All-cause mortality at 5 and 10 years ● Graft survival at 5 and 10 years
Kasiske *et al*. [16] (2003)	Retrospective cohort	Renal transplant recipients (*n* = 11 659) ● No diabetes at 36 months (76.0%) ● PTDM at 36 months (24.0%)	● Patients in the USRDS who received their first kidney transplant in 1996–2000 ● Patients who had Medicare as their primary payer	● Patients with other organ transplants ● Patients without Medicare as their primary payer ● Patients with employer group health insurance ● Diabetes at the time of transplantation ● Patients who were treated with improbable drug combinations	● All-cause mortality ● Cardiovascular mortality ● Sepsis mortality ● Malignancy mortality ● Graft survival ● DCGL
Khalkhali *et al*. [47] (2010)	Retrospective cohort	Renal transplant recipients (*n* = 1534)	● Renal transplant recipients at a single center from 1997 to 2005	N/A	● DCGL
Kumar *et al*. [70] (2020)	Prospective cohort	Total living-donor renal transplant recipients (*n* = 100) ● No diabetes (*n* = 76) ● PTDM (*n* = 24)	● Adult patients with ESRD who underwent live donor kidney transplantation ● Absence of diabetes prior to kidney transplantation ● Received standard triple immunosuppressive medications ● Patients who were capable of understanding the study and given informed written consent for study participation	● Patients with a diagnosis of DM prior to kidney transplantation ● Patients who received anti-diabetic medication ● Patients who did not give consent for the study	● All-cause mortality ● Graft survival
Lim *et al*. [18] (2021)	Retrospective cohort	1976 living-donor, 3196 deceased-donor renal transplant recipients (*n* = 5248) ● No diabetes (*n* = 2476) ● PTDM (*n* = 799) ● Pre-existing diabetes (*n* = 1973)	● Adult kidney transplant recipients registered at Canadian Organ Replacement Register	● Individuals aged <18 or >105 years at time of transplant ● Had an invalid or no recorded date of birth or sex ● Date of death on or before the index date ● Non-Ontario residents ● Prior history of solid organ transplant	● All-cause mortality at 0–5, 5–9 and >9 years ● Cardiovascular mortality
Lv *et al*. [67] (2014)	Retrospective cohort	101 living-donor, 328 deceased-donor renal transplant recipients (*n* = 429) ● No diabetes (*n* = 342) ● PTDM (*n* = 87)	• Patients who underwent a renal transplant, from January 1993 to December 2008 in a single center	• Unclear data on preoperative medical history • Missing postoperative follow-up information • Patients whose renal graft survived <1 year after surgery • Patients with multi-organ transplant • Patients who underwent 2 or more renal transplants • Patients who were diabetic before surgery	● All-cause mortality at 5 and 10 years
Maekawa *et al*. [73] (2020)	Retrospective cohort	Total living-donor renal transplant recipients (*n* = 220) ● No PTDM (*n* = 190) ● PTDM (*n* = 30)	● Patients with ESRD who underwent kidney transplant from living donors ● Patients followed for at least 1 year after surgery	● Deceased-donor transplants ● Missing data ● Patients who received cyclophosphamide, methotrexate or azathioprine, and had a history of cancer before transplant	● All-cause mortality at 5 and 10 years
Malik *et al*. [55] (2021)	Prospective cohort	498 living-donor, 134 deceased-donor renal transplant recipients (*n* = 632) ● No diabetes (*n* = 446) ● PTDM (*n* = 186)	● Kidney recipients from 13 participating transplant centers	● Patients with pre-transplant diabetes ● Recipient age <16 years ● Donor age <5 years ● Missing covariate data ● En-bloc transplant	● All-cause mortality ● Graft survival ● DCGL
Miles *et al*. [51] (1998)	Retrospective cohort	12 living-donor, 66 deceased-donor renal transplant recipients (*n* = 78) ● Control (*n* = 38) ● PTDM (*n* = 40)	● Patients whose grafts survived for at least 1 year ● Patients with normal FBG levels before transplantation	N/A	● All-cause mortality ● Cardiovascular mortality ● Sepsis mortality ● Malignancy mortality ● Graft survival
Nagaraja *et al*. [45] (2013)	Retrospective cohort	Total deceased-donor renal transplant recipients (*n* = 118) ● No diabetes (*n* = 74) ● PTDM (*n* = 44)	● Age 18–80 years ● No history of type 2 DM and fasting glucose <7.0 mmol/L on at least two occasions in the year before transplantation	N/A	● All-cause mortality ● Cardiovascular mortality ● Sepsis mortality ● Malignancy mortality
Nagib *et al*. [63] (2015)	Case–control	Total living-donor renal transplant recipients (*n* = 906) ● No diabetes (*n* = 456) ● PTDM (*n* = 450)	● Patients who received live-donor renal allograft in a single center between March 1976 and November 2010	N/A	● All-cause mortality at 8 and 15 years ● Graft survival
Nie *et al*. [29] (2019)	Retrospective cohort	Renal transplant recipients (*n* = 96 669) ● No PTDM (*n* = 96 538) ● PTDM (*n* = 131)	● ICD-9 diagnostic code indicating renal transplantation status more than 1 year earlier	● Patients with the diagnosis of diabetes for more than 1 year prior to renal transplantation	● Graft survival
Ouni *et al*. [64] (2022)	Retrospective cohort	247 living-donor, 10 deceased-donor kidney transplant recipients (*n* = 257) ● No diabetes (*n* = 201) ● PTDM (*n* = 56)	● Patients who underwent their first kidney transplant between 2008 and 2019	● DM before transplant	● All-cause mortality ● Graft survival
Park *et al*. [43] (2015)	Retrospective cohort	A total of 176 kidney transplant recipients ● 58 had transient hyperglycemia ● 48 developed PTDM	● Kidney transplant recipients without a known history of DM ● Patients who underwent kidney transplantation at Kyungpook National University Hospital between August 2001 and June 2012 ● Patients who were followed up for at least 1 year after transplantation	● Patients younger than 18 years old ● Patients diagnosed with DM before transplantation	● All-cause mortality
Porrini *et al*. [42] (2019)	Retrospective cohort	603 renal transplant recipients: ● PTDM (*n* = 98) ● Normal (*n* = 342) ● Prediabetes (*n* = 163)	● Renal transplant recipients who underwent transplantation ● Patients followed up for a median of 8 years	N/A	● All-cause mortality
Romagnoli *et al*. [60] (2005)	Case–control	Consecutive renal transplant recipients who underwent kidney transplantation (*n* = 538) • A pair-matched analysis design was utilized (*n* = 32 pair-matched controls)	● Renal transplant recipients treated with different immunosuppressive regimens ● Patients who underwent kidney transplantation at the Catholic University of Rome, Italy, during the specified period ● Patients with available data on baseline immunosuppression and the development of PTDM	N/A	● All-cause mortality ● Graft survival
Rosettenstein *et al*. [28] (2016)	Prospective cohort	Nondiabetic kidney transplant recipients (*n* = 83): ● PTDM (*n* = 14) ● Normal (*n* = 43) ● IFG or IGT (*n* = 26)	● Incident nondiabetic (no prior history of pretransplant diabetes with normal-range FBG and random blood glucose levels before transplantation) live and deceased donor ABO-compatible kidney transplant recipients ● Transplanted between 2008 and 2011	● Recipients with known pretransplant diabetes or abnormal FBG and random blood glucose levels before transplantation	● Graft survival
Roth *et al*. [50] (1989)	Retrospective cohort	Nondiabetic kidney transplant recipients (*n* = 314): • PTDM (*n* = 49) • No diabetes (*n* = 265)	• Patients who underwent renal transplantation between January 1979 and June 1987 at a single center	• Patients with known DM • Patients with early graft failure	● All-cause mortality ● Graft survival
Savaj *et al*. [71] (2008)	Retrospective cohort	Kidney transplant recipients with a negative history of DM before transplantation (*n* = 203) ● PTDM (*n* = 39) ● No diabetes (*n* = 164)	● Patients who underwent renal transplantation from January 2001 to March 2005 at a single center	N/A	● All-cause mortality ● Graft survival
Sezer *et al*. [59] (2006)	Retrospective cohort	Patients who underwent renal transplantation (*n* = 204) ● PTDM (*n* = 26) ● Non-PTDM (*n* = 178)	● Patients who underwent renal transplantation between 1996 and 2002 ● Minimum follow-up of 30 months post transplantation	● Patients with known DM prior to transplantation ● Patients diagnosed with DM within 2 months post-transplantation	● Graft survival
Sharma *et al*. [69] (2003)	Case–control	Total patients (*n* = 1023): ● PTDM group (*n* = 100) ● Control group: 100 paired nondiabetic renal transplant patients matched for sex and transplantation date	● Patients who underwent renal transplantation between January 1989 and June 2000 ● Patients without preexisting DM	● Patients with preexisting DM	● All-cause mortality ● Graft survival
Sheu *et al*. [38] (2016)	Retrospective cohort	Total number of patients analyzed (*n* = 148) ● Patients with preexisting DM (*n* = 29) ● Patients who developed PTDM (*n* = 27) ● Patients without DM or PTDM (non-diabetic group) (*n* = 92)	● Adult recipients of renal transplants from a single center over 5.5 years ● Patients with at least 1 year of follow-up data available	N/A	● All-cause mortality
Silva *et al*. [58] (2000)	Retrospective cohort	Total number of patients analyzed (*n* = 825) ● Patients who developed PTDM (*n* = 33) ● Control patients without PTDM (*n* = 28)	● Patients who underwent renal transplantation between 1985 and 1988 ● Patients whose grafts survived for at least 1 year ● Patients with normal FBG levels before transplantation	● Patients who underwent renal transplantation between 1985 and 1988 ● Patients whose grafts survived for at least 1 year ● Patients with normal FBG levels before transplantation	● All-cause mortality ● Cardiovascular mortality ● Sepsis mortality ● Malignancy mortality
Siraj *et al*. [52] (2010)	Case–control	Patients who received single-organ kidney transplant (*n* = 94) • PTDM (*n* = 47) • No diabetes (*n* = 47)	● Patients who received single-organ kidney transplantation at a single center between 1996 and 1998	● Multi-organ transplants	● All-cause mortality ● Cardiovascular mortality
Sulanc *et al*. [46] (2005)	Retrospective cohort	Nondiabetic patients who received kidney transplantation (*n* = 122) • PTDM (*n* = 80) • No diabetes (*n* = 42)	● Consecutive nondiabetic patients who received kidney transplantation between August 2001 and March 2003 at a single center	● Younger than 19 years old ● Diagnosed with DM prior to transplantation ● Elevated FBG before the time of the transplant ● Multiple organ transplantation	● All-cause mortality ● Cardiovascular mortality
Tsai *et al*. [35] (2011)	Retrospective cohort	Renal transplant recipients (*n* = 427) • Pre-transplant diabetes (*n* = 70) • PTDM (*n* = 104) • No diabetes (*n* = 253)	● Patients who had undergone renal transplantation from December 1999 to January 2008	N/A	● All-cause mortality ● Graft survival
Tutone *et al*. [32] (2004)	Retrospective cohort	A total of 1186 patients ● Type 1 diabetes (*n* = 91) ● Type 2 diabetes (*n* = 8) ● PTDM (*n* = 93)	● First-time recipients of either cadaveric or living-donor renal transplants ● Patients with available data on random blood glucose measurements and other relevant clinical parameters ● Patients with diabetic nephropathy or PTDM were included in the study	● Subsequent renal transplants in patients whose first graft failed were not included in the analysis ● Patients with incomplete data on outcomes were excluded from certain analyses	● All-cause mortality ● Graft survival
Veroux *et al*. [53] (2013)	Retrospective cohort	Total population (*n* = 436) ● Number of individuals with PTDM (*n* = 29) ● Individuals without PTDM (*n* = 407)	● Patients with ESRD who received kidney transplantation at the Organ Transplant Unit of the University Hospital of Catania between January 2001 and April 2008 ● Patients with a diagnosis of PTDM	● Patients with a diagnosis of DM as a cause of ESRD	● All-cause mortality ● Graft survival
Wauters *et al*. [56] (2012)	Retrospective cohort	Total population: 1146 adults ● Patients PTDM (*n* = 153) ● Patients without PTDM (*n* = 651) ● IFG (*n* = 342)	● Adults who received their first kidney transplant between 1984 and 2008 ● Patients treated with modern immunosuppressants	● Prior bone marrow/solid organ transplant recipients ● Pre-existing diabetes (FPG ≥126 mg/dL or on glucose-lowering drugs)	● All-cause mortality ● Cardiovascular mortality ● Sepsis mortality ● Malignancy mortality
Yeh *et al*. [39] (2020)	Retrospective cohort	A total of 3663 kidney transplant recipients: ● Pre-existing DM before transplantation (*n* = 531) ● PTDM after transplantation (*n* = 631) ● Recipients did not have DM (non-DM group) (*n* = 2501)	● Patients who received kidney transplantation between 1 January 1998 and 31 December 2011, in Taiwan ● Patients with available data in the NHIRD for analysis ● Patients aged 20 years or older at the time of transplantation	● Patients who died within 3 months after transplantation ● Patients newly diagnosed with diabetes within 30 days after transplantation ● Patients with missing demographic data ● Patients younger than 20 years old	● All-cause mortality ● Cardiovascular mortality ● Sepsis mortality ● DCGL

DBD, donation after brain death; DCD, donation after circulatory death; DCGL, death-censored graft loss; DM, diabetes mellitus; ESRD, end-stage renal disease; ICD-9, International Classification of Diseases, Ninth Revison; IFG, impaired fasting glucose; N/A, not applicable; NHIRD, National Health Insurance Research Database; USRDS, United States Renal Data System.

The type of donor was very different between the included studies. Although 14 trials did not specify the exact percentages [16, 29–31, 35, 39, 41, 42, 47, 49, 57, 60, 66, 69], the percentage of deceased donors varied between 0% (only living donors) in 6 [34, 62, 63, 65, 70, 73] and 100% (only deceased donors) in 6 manuscripts [44, 45, 48, 55, 58, 61], with the majority of the studies having more than 50% of the grafts from deceased donors [18, 27, 28, 32, 33, 37, 38, 40, 44–46, 48, 50–55, 58, 61, 67, 68, 75–77].

PTDM definition was inconsistent across the studies. One study included patients with fasting blood glucose (FBG) ≥150 mg/dL [51]. Two trials considered PTDM patients who previously were not diabetic, but required treatment of hyperglycemia [31, 44]. Two studies used an FBG ≥120 mg/dL and a 2 h post-prandial blood glucose ≥200 mg/dL [30, 74], while three other ones used random glucose measurements ≥200 mg/dL [32–34]. Tutone *et al*. and Joss *et al*. also considered the use of hypoglycemic treatment [32, 33]. Five studies included those with an FBG ≥140 mg/dL: two used only this criterion [50, 58], while the other three considered also the use of hypoglycemic medication [9, 26, 68].

The majority of the included papers considered an FBG ≥126 mg/dL. Two used only this standard [36, 60], one additionally an oral glucose tolerance test (OGTT) ≥200 mg/dL or treatment [72], four considered also hypoglycemic treatment [41, 42, 45, 56] and six also a random blood glucose ≥200 mg/dL [49, 52, 53, 65, 66, 76]. Cheng *et al*. included also patients on diet or on specific treatment [49], while five studies assessed also with a 2 h post-prandial blood glucose (or an OGTT) ≥200 mg/dL [28, 61, 62, 69, 71]. Ten other studies, aside the FBG ≥126 mg/dL, further used a 2-h post-prandial blood glucose (or OGTT) ≥200 mg/dL or a random blood glucose ≥200 mg/dL [35, 37, 38, 46, 48, 54, 63, 64, 67, 73], while Hussain *et al*. considered PTDM patients with an FBG ≥126 mg/dL or OGTT ≥ 200 mg/dL or a random blood glucose ≥200 mg/dL or hemoglobin A1c (HbA1c) levels ≥6.5% [40]. Two studies included in the analysis patients with HbA1c levels ≥6.5%, random blood glucose levels (or OGTT) ≥200 mg/dL, or the prescription of any antidiabetic medication [43, 70]. Finally, five studies used documentation of diabetes in the electronic health record [16, 18, 29, 39, 55], and two did not specify the definition [57, 77].

### All-cause mortality

**Figure 2:**
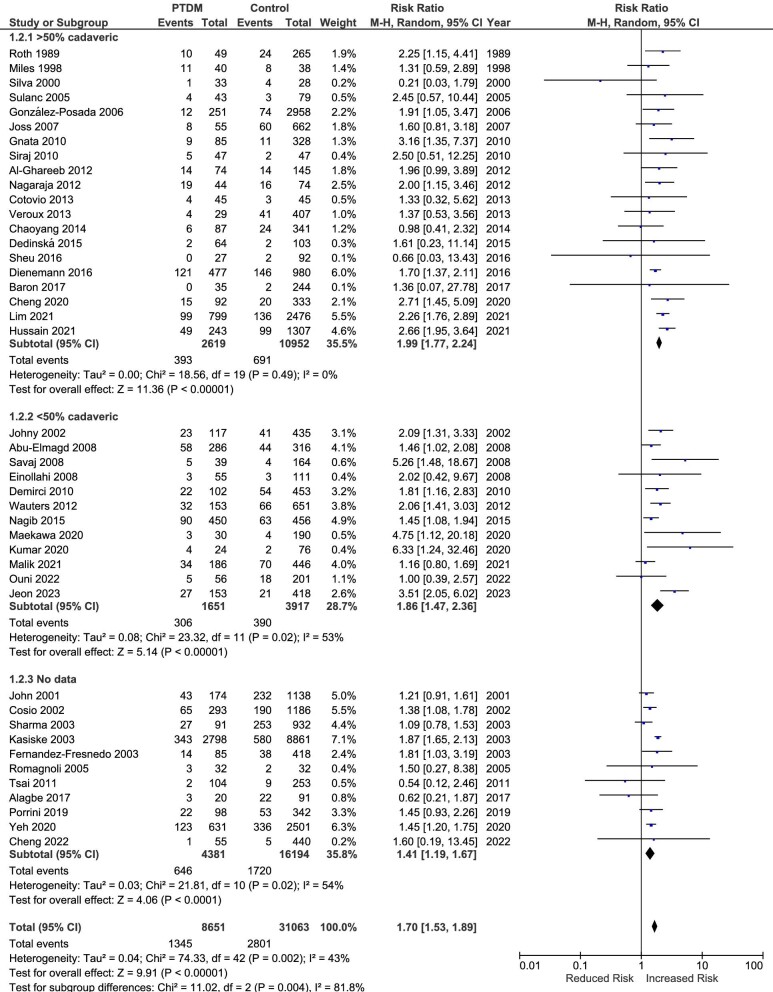
Meta-analysis of PTDM and the risk of all-cause mortality.

There were 46 studies that analyzed the relationship between the development of PTDM and all-cause mortality [9, 16, 27, 30–35, 37–40, 18, 42–46, 48–58, 60, 62–76]. Eight studies presented the results at different follow-up times: at 5 and 10 years [9, 33, 67, 73, 75], at 8 and 15 years [62, 63], and at 0–5, 5–9 and >9 years [18]. As shown in Fig. 2, PTDM was significantly associated with an increased risk for all-cause mortality (RR 1.70, 95% CI 1.53 to 1.89, *P* < .001; heterogeneity χ^2^ = 74.33, I^2^ = 43%, *P* = .002). Using the most distal follow-up time for the eight studies mentioned above did not change the main estimates, but increased the heterogeneity (RR 1.82, 95% CI 1.55 to 2.14, *P* < .001; heterogeneity χ^2^ = 255.31, I^2^ = 84%, *P* < .001) (Supplementary data, Fig. S1). Four studies were not included in the meta-analysis. In line with our results, Tutone *et al*. [32] stated that PTDM had an adverse effect on patient survival (*P* < .001, log-rank test). By contrast, Park *et al*. mentioned that there was no difference in the incidence of patient death between the analyzed groups [43]. In the other two studies, there were no deaths during the follow-up, so no estimates could be computed [44, 65].

Given the increased heterogeneity, we performed a sensitivity analysis focusing on the type of donor (>50% and <50% cadaveric donors); studies that did not mention the type of donor were included in a different group. As observed in Fig. 2, there was a significant difference in the association between PTDM and this outcome between the three groups of studies, with an increased risk in patients from studies with more than 50% cadaveric donors. Interestingly, the heterogeneity in this group of studies was minimal (I^2^ = 0%).

### Cardiovascular mortality

**Figure 3:**
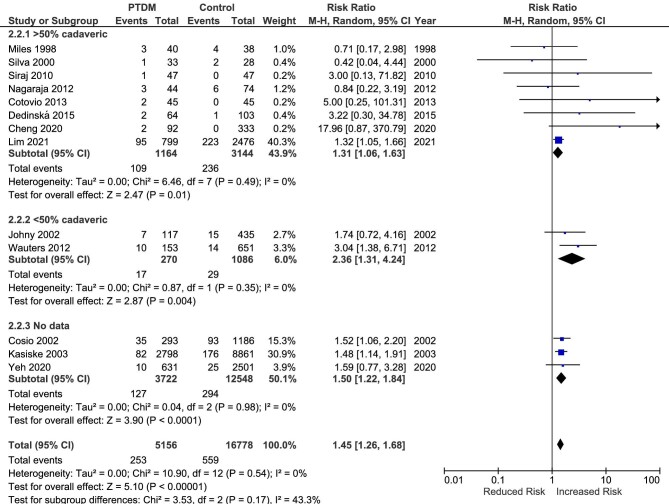
Meta-analysis of PTDM and the risk of cardiovascular mortality.

Cardiovascular mortality was evaluated in 14 studies [16, 31, 39, 18, 45, 46, 48, 51, 52, 56, 58, 61, 68, 74]. Overall, there was a significant increase in the risk for cardiovascular mortality associated with PTDM (RR 1.86, 95% CI 1.36 to 2.54, *P* < .001; heterogeneity χ^2^ = 21.1, I^2^ = 43%, *P* = .05; Fig. 3). In the sensitivity analysis, there were no significant differences between the three groups of studies.

### Sepsis mortality

**Figure 4:**
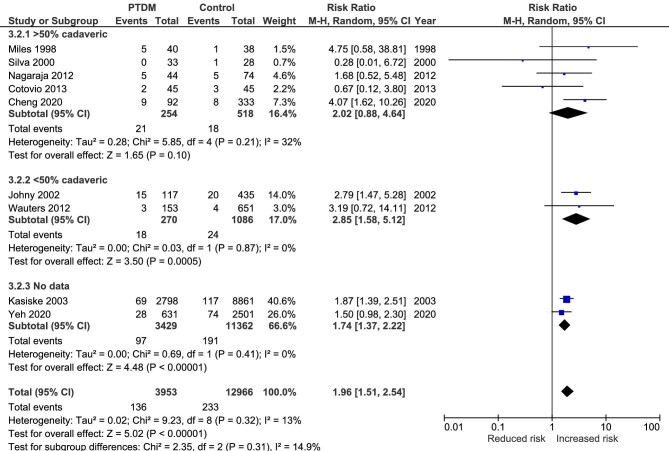
Meta-analysis of PTDM and the risk of sepsis-related mortality.

Nine studies assessed the relationship between PTDM and sepsis mortality [16, 39, 45, 48, 51, 56, 58, 68, 74]. As shown in Fig. 4, there was a significant increase in the risk for sepsis mortality associated with the presence of PTDM (RR 1.96, 95% CI 1.51 to 2.54, *P* < .001; heterogeneity χ^2^ = 9.23, I^2^ = 13%, *P* = .32). Similar to the previous outcome, there were no differences between the three groups.

### Malignancy mortality

**Figure 5:**
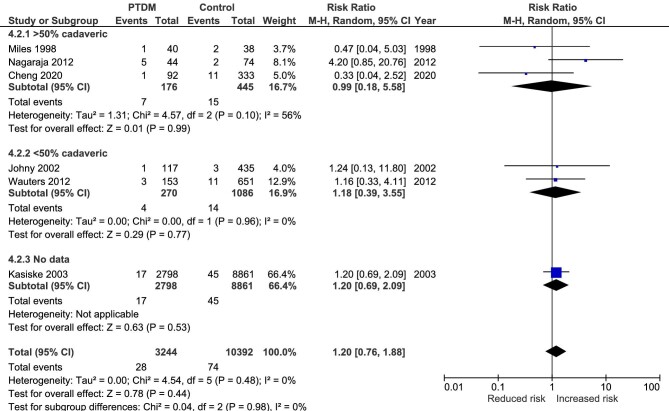
Meta-analysis of PTDM and the risk of malignancy-related mortality.

This outcome was analyzed in seven studies [16, 45, 51, 56, 58, 68, 74]. There was no significant association between PTDM and malignancy mortality (RR 1.20, 95% CI 0.76 to 1.88, *P* = .44; heterogeneity χ^2^ = 4.54, I^2^ = 0%, *P* = .48; see Fig. 5). There were no malignancy deaths in the paper by Silva *et al*. [58].

### Graft survival

**Figure 6:**
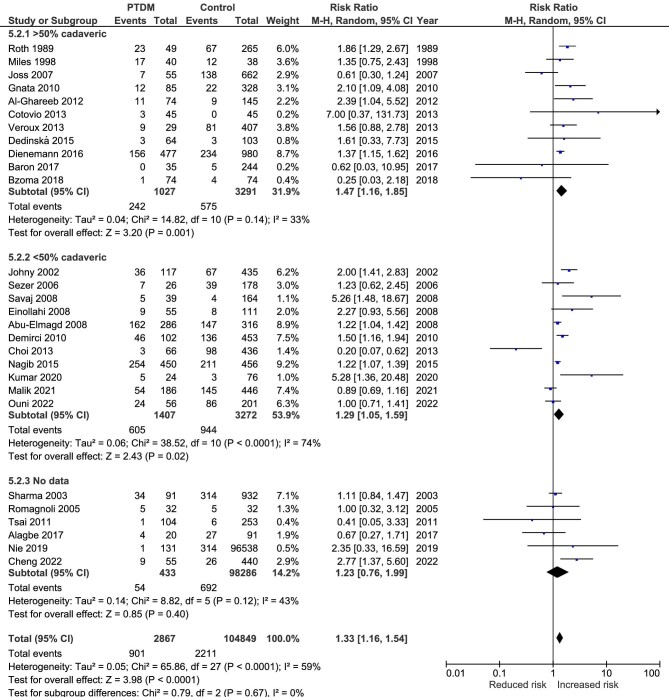
Meta-analysis of PTDM and the risk of graft loss.

Graft survival was an outcome in 36 studies [9, 16, 26, 28, 29, 32–37, 39, 44, 47–51, 53–55, 59–66, 69–72, 74–76]. Three studies presented the results at different follow-up times: at 5 and 10 years [33, 36, 75], five showed the results for both overall and death-censored graft loss [9, 16, 37, 55, 72], while three presented only for death-censored graft loss [26, 39, 47]. Figure 6 shows that PTDM is associated with an increased risk for overall graft failure (RR 1.33, 95% CI 1.16 to 1.54, *P* < .001; heterogeneity χ^2^ = 65.86, I^2^ = 59%, *P* < .001). Since four studies [16, 26, 39, 72] showed only the HRs (without the exact numbers for graft survival) we performed an additional analysis, shown in Supplementary data, Fig. S2 (HR 1.55, 95% CI 1.33 to 1.81, *P* < .001; heterogeneity χ^2^ = 6.97, I^2^ = 57%, *P* = .07). Changing the duration of the follow-up or the type of graft loss did not alter the direction and the statistical significance of this relationship (data not shown). Four studies were not included in the meta-analysis. Two studies mentioned that there was no association between PTDM and this outcome [32, 47], while in the other two studies, there were no graft losses during the follow-up, so no estimates could be computed [28, 65].

In the sensitivity analysis, there were no significant differences between the three groups of patients regarding the association between PTDM and the outcome; the heterogeneity was increased only in the group of studies with <50% cadaveric donors (Fig. 6).

## DISCUSSION

Kidney transplant recipients with PTDM face a significantly higher risk of all-cause mortality compared with non-PTDM patients, exhibiting a RR of 1.70 (95% CI 1.53 to 1.89, *P* < .001). Other studies have reported similar results. For example, a meta-analysis of 14 retrospective studies involving 9872 PTDM patients and 65 327 non-diabetic recipients revealed a 67% increase in all-cause mortality risk and a 35% increase in graft failure risk among PTDM patients [22]. Moreover, a single-center analysis of 1990 kidney recipients found that PTDM treated with medication at 1 year had comparable risks of overall mortality and death with functioning graft [78], emphasizing the ongoing significance of PTDM as a risk factor despite proper medical intervention. Another retrospective cohort study of 959 solid organ transplant recipients indicated that PTDM patients had 89% higher risk of all-cause mortality compared with non-diabetic patients, showcasing the overall solid transplant patients under similar risk to our particular renal transplant group [79]. Finally, a study involving 3782 kidney transplant patients experiencing allograft loss reported that those with PTDM had about 50% higher risks of mortality on dialysis compared with those without diabetes [80], highlighting the long-term ramifications of PTDM on patient outcomes post-rejection; this study emphasizes the enduring effects of PTDM, signaling its persistence beyond the transplantation phase. However, there are oppositions related to the aforementioned findings. To illustrate, in a multicenter cohort study of 632 nondiabetic adult kidney transplant recipients, there was no significant association between PTDM and mortality over a median follow-up of 6 years [55]. In another retrospective cohort study of 571 kidney transplant recipients, PTDM did not significantly impact mortality rates or MACE compared with non-diabetic recipients [72]. PTDM was also not found to be a predictor of all-cause mortality within the first 8 years after kidney transplantation [17]. Other studies have reported similar results [81, 82]. Nevertheless, despite the mentioned opposition by some studies, the overall results from this study and previous meta-analyses support an elevated risk of mortality faced by PTDM patients [22].

In our study, we conducted an in-depth investigation into cardiovascular mortality, a facet often overlooked in comparable research endeavors. Our findings revealed a notable increase in the risk of cardiovascular mortality among PTDM patients, as evidenced by an 86% increased relative risk albeit with moderate heterogeneity. The correlation between cardiovascular mortality and PTDM is particularly significant, given ample evidence implicating endothelial dysfunction, damage and subsequent plaque formation in this relationship [83]. Although PTDM and type 2 diabetes are distinct entities, both may contribute to MACE due to shared risk factors [83]. Numerous studies have consistently demonstrated a significant association between dysglycemia and adverse cardiovascular outcomes. For instance, a study involving 490 kidney transplant recipients revealed that individuals with fasting glucose levels >100 mg/dL exhibited a notably higher incidence of cardiac and peripheral vascular disease events [84]. Similarly, another study found that patients with PTDM exhibited more than 3-fold higher risk of MACE compared with their nondiabetic counterparts [17]. Further substantiating these findings, a study involving 7612 kidney transplant recipients in South Korea also demonstrated a significantly elevated risk of MACE among PTDM patients [85]. In another study involving 3663 kidney transplant recipients, PTDM patients displayed increased risks of MACE, all-cause mortality and death with functioning grafts, particularly among individuals younger than 55 years old [86]. However, conflicting results exist, potentially attributable to factors such as small sample sizes or selection biases. For instance, a retrospective cohort study comprising 571 kidney transplant recipients did not reveal a significantly elevated risk of death or MACE in the PTDM group compared with the non-diabetes mellitus group [72]. In summary, our study highlights a significant association between PTDM and increased cardiovascular mortality.

In the sepsis mortality analysis, individuals with PTDM exhibited a nearly 2-fold greater likelihood of dying from sepsis compared with their non-PTDM counterparts. In contrast, PTDM was not associated with higher risk of death due to malignancies. Sepsis has previously been reported to increase the risk of mortality and graft loss among kidney transplant recipients [87]. Indeed, among patients with diabetes at the time of allograft loss, infection-related mortality increased substantially with an adjusted subdistribution HR of 2.70 for PTDM [80].

Our findings show a substantial risk of graft loss among PTDM patients with a 33% higher risk. This disparity underscores the deleterious impact of PTDM on graft survival. PTDM engenders a host of challenges, notably stemming from issues with immunosuppressive regimens [88]. Non-adherence to these regimens poses a significant threat to graft viability. Moreover, the complexity of immunosuppressive treatments necessitates modifications, further complicating management and potentially compromising graft integrity [83]. Metabolic consequences associated with PTDM further exacerbate the risk of graft loss [83]. Other studies underscore the pivotal role of PTDM in graft viability following kidney transplantation. For instance, among 2749 adult Norwegian renal transplant recipients, individuals afflicted with PTDM exhibited a 50% higher risk for graft loss, while the risk for death-censored graft loss was 25% higher when compared with recipients without PTDM [82]. Another investigation demonstrated that kidney transplant recipients without PTDM had 5- and 10-year graft survival rates of 97.8% and 96.0%, respectively, versus recipients with PTDM who had corresponding graft survival rates of 90% and 63% [89]. A retrospective analysis encompassing 939 renal transplant recipients found that patients with PTDM exhibited a mean graft survival period of 9.7 years, in contrast to 11.3 years observed in non-diabetic counterparts [90].

Although direct investigations into the associations of PTDM with mortality and graft loss are scarce, several plausible mechanisms are recognized. Endothelial damage may lead to vascular injury that compromises perfusion of the kidney, heart and other organs. Moreover, burgeoning evidence underscores dysglycemia as a condition fostering a milieu of chronic inflammation, a risk factor for chronic kidney disease and atherosclerosis [91]. Assessments focusing of immune function including cell adherence, chemotaxis, phagocytosis and bactericidal activity have consistently revealed diminished performance in diabetic individuals [92, 93]. PTDM combined with immunosuppression renders patients vulnerable to bacteremia and its sequelae [94].

### Limitations and strengths

The analysis presents several limitations that warrant consideration. First, the heterogeneity in follow-up periods among the included studies introduces variability and potential biases in the results. Additionally, the global distribution of the studies introduces variability due to differences in healthcare systems and patient populations. Publication bias is another concern, as studies with significant results may be overrepresented, impacting the overall conclusions. Moreover, inconsistencies in reporting and the exclusion of some studies due to insufficient data or lack of events during follow-up may limit the comprehensiveness of the analysis. Furthermore, the assessment of outcomes was limited to specific endpoints, potentially overlooking other relevant factors related to PTDM outcomes. Finally, the analysis may not have accounted for all confounders, such as immunosuppressive regimens or donor characteristics, which could influence the observed associations. In addressing the observed significant heterogeneity in our meta-analysis, it becomes evident that several contributing factors could account for this variability. Firstly, variations in the definitions and diagnostic criteria of PTDM across studies may lead to differing inclusion criteria and thus influence the pooled outcomes. Moreover, the diversity in immunosuppressive regimens employed post-transplantation is known to affect metabolic parameters, potentially contributing to the observed heterogeneity. Additionally, the inherent differences in patient demographics, such as age, ethnicity and comorbidities, as well as graft characteristics including donor source, could further introduce variability into our findings. These multifaceted aspects underline the complexity of PTDM epidemiology and necessitate a nuanced interpretation of our results in the context of these diverse contributing factors. Addressing these points not only highlights the breadth of factors influencing PTDM outcomes but also underscores the importance of future research efforts aimed at standardizing definitions and refining therapeutic approaches to mitigate heterogeneity and improve clinical management strategies for transplant recipients.

Despite these limitations, the analysis possesses several strengths. The inclusion of a substantial number of studies and patients provides a robust basis for assessing the relationship between PTDM and post-transplant outcomes. The meta-analysis approach allows for the synthesis of data from multiple studies, enhancing statistical power and providing generally reliable estimates of effect size. Moreover, the analysis systematically evaluated outcomes such as all-cause mortality, cardiovascular mortality, sepsis mortality, malignancy mortality and graft survival, providing a comprehensive understanding of PTDM's impact on transplant recipients. Additionally, the analysis highlights consistencies across studies, reinforcing the validity of the findings.

## CONCLUSION

In our systematic review and meta-analysis encompassing 53 studies and 138 917 patients, we found evidence linking PTDM to increased risks of all-cause mortality, cardiovascular mortality and sepsis mortality. Furthermore, PTDM emerged as a significant factor contributing to a heightened risk of graft failure. In light of these consequential findings underscoring the prognostic impact of PTDM, it is paramount to prioritize regular monitoring for onset of diabetes during the post-transplant phase to enable timely diagnosis and management. Furthermore, the potential underrepresentation of reported PTDM in transplant registries underscores the necessity for improved methodologies for comprehensive assessment, to better discern its impact on transplant outcomes. The findings of our study shed light on the profound impact of PTDM on patient prognosis and underscore the importance of not only early detection but also effective and timely management of this condition. Consequently, further research is crucial to develop optimal management protocols and regimens involving suitable glucose-lowering agents for PTDM. Given the established connections between PTDM and patient prognosis, particularly in light of our findings indicating an elevated risk of cardiovascular mortality, all-cause mortality and overall graft loss among PTDM patients, it is imperative to focus on investigating Sodium-Glucose Cotransporter 2 (SGLT-2) inhibitors and Glucagon-Like Peptide-1 (GLP-1) receptor agonists, as these agents warrant special attention due to their potential renoprotective and cardioprotective effects, as well as their known ability to improve overall mortality.

## Supplementary Material

gfae185_Supplemental_Files

## Data Availability

The data is available upon request.
