# Supplementary material for: Prognostic impact of post-transplant diabetes mellitus in kidney allograft recipients: a meta-analysis
Source: Nephrol Dial Transplant. 2024 Aug 12;40(3):554–76. doi: 10.1093/ndt/gfae185 (PMC11879034; doi:10.1093/ndt/gfae185)
Supplement: gfae185_Supplemental_Files [file gfae185_supplemental_files.zip › Supplementary Table 1.docx]

**Supplementary Table S3**. Definitions of the cardiovascular mortality in the studies analyzed.

| **Author (publication year)** | **Definition of cardiovascular mortality** |
| --- | --- |
| **Description**: The cardiovascular outcomes reported in the following studies were included. Rather than having a specific predefined classification, some studies with limited sample sizes employed reporting of individual outcomes in the text. For our meta-analysis, we included the deaths that occurred due to myocardial infarction, cardiac arrest, sudden cardiac death, and cerebrovascular events including stroke or non-specified events, to assess cardiovascular death. | |
| Miles (1998) | Mortality was further classified into sepsis, cardiovascular, malignancy, and other causes. No specific details regarding the definition of cardiovascular mortality have been provided. |
| Silva (2000) | Causes of mortality were classified into the following based on the reported cases within the patient group:   1. Colic perforation 2. Cardiovascular 3. Malignancy 4. Other   No further specific definition exists. Only the cardiovascular category was included in cardiovascular deaths. |
| Cosio (2002) | The patients are classified according to the cause of death. Most of the patients were marked as unknown. Cardiovascular deaths were the only specified group without further definition. |
| Johny (2002) | Causes of death for all patients were subgrouped into:   1. Infections 2. Coronary heart disease 3. Stroke 4. Malignancy 5. Liver related 6. Surgical   The number of patients who died due to coronary heart disease and stroke were selected and combined to be used in our study. |
| Kasiske (2003) | The authors classified deaths into specific causes:   1. Cardiovascular 2. Malignancy 3. Infection 4. Unknown causes   Only the numbers for cardiovascular deaths are included in this specific analysis. |
| Siraj (2010) | Only one patient deceased due to specified cardiovascular complications. The patient was specifically referred to as “cardiovascular death” in the article. |
| Nagaraja (2012) | The article used the following statement:  “Cardiovascular cause of death was defined as death  because of myocardial infarction, cerebral haemorrhage,  cerebral infarct or cardiac arrest because of unknown cause.” |
| Wauters (2012) | Cardiovascular deaths are sub-specified as deaths occurring due to cardiac events. The definition of the individual events are defined in the following way:  “Pretransplantation and posttransplantation MCVE (major cardiovascular events) were defined as fol-  lows: Cardiac events included acute myocardial infarction and coronary revascularization either surgical or percutaneous (angioplasty or stents); vascular events included amputations, revascularization for peripheral vascular disease either surgical or percutaneous, and also central vascular events such as abdominal aortic aneurysm and carotid artery or renal artery stenosis requiring intervention; CNS events included all cerebrovascular accidents.” |
| Cotovio (2013) | 2 patients were reported to be deceased due to cardiovascular disease. In the study:   - “Cardiovascular disease (CVD) definition included coronary, cerebrovascular, peripheral artery disease and/or congestive heart failure. - “Acute cardiovascular events definition included acute myocardial infarction, stroke, and acute heart failure.” |
| Dedinska (2015) | The definition was not mentioned. |
| Cheng (2020) | Causes of patient death were subgrouped:   1. Infection 8 (40.0%) 9 (60.0%) 2. Cancer 11 (55.0%) 1 (6.7%) 3. Hepatic failure 0 (0.0%) 2 (13.3%) 4. Cardiovascular sudden death 0 (0.0%) 1 (6.7%) 5. CVA 0 (0.0%) 1 (6.7%) 6. Others 1 (5.0%) 1 (6.7%)   Cardiovascular sudden death and cerebrovascular accident (CVA) were included. |
| Yeh (2020) | Mortality was further classified into sepsis, cardiovascular, malignancy, and other causes. The rationale behind the classification into cardiovascular disease-related mortality and other specific causes of mortality has been justified in the following way:  “These outcomes were detected based on the principal or secondary diagnosis of an emergency department visit or hospitalization. Components of cardiovascular diseases were detected based on the principal diagnosis of any emergency visit or hospitalization, most of these diagnostic codes for which have been previously validated.” |
| Lim (2021) | The diagnostic codes were provided in the following supplementary link: <http://links.lww.com/TP/C119>  Based on The Office of the Registrar General (ORGD), the following codes were used to define cardiac mortality:  LCD_33, LCD_34, LCD_35, LCD_36, LCD_37, LCD_38, LCD_39,  LCD_40, LCD_41, LCD_42, LCD_43, LCD_44 |
