# Supplementary material for: Prognostic impact of post-transplant diabetes mellitus in kidney allograft recipients: a meta-analysis
Source: Nephrol Dial Transplant. 2024 Aug 12;40(3):554–76. doi: 10.1093/ndt/gfae185 (PMC11879034; doi:10.1093/ndt/gfae185)
Supplement: gfae185_Supplemental_Files [file gfae185_supplemental_files.zip › Supplementary Table 2.docx]

**Supplementary Table S1. Detailed search strategy and key search terms**

| **PubMed (n=913)** |
| --- |
| 4 (("Kidney Transplantation"[Mesh] OR "Kidney Transplantation" OR "Renal Transplantation" OR "Kidney Transplantations" OR "Kidney Grafting") AND ("new onset diabetes" OR "posttransplant diabetes mellitus" OR "posttransplant diabetes" OR "post-transplant diabetes mellitus" OR "post-transplant diabetes")) AND ("Prognosis"[Mesh] OR prognoses OR prognosis OR "prognostic factor" OR "Mortality"[Mesh] OR mortality OR mortalities OR "Survival"[Mesh] OR survival OR "Graft Survival"[Mesh] OR "graft survival" OR "graft survivals" OR "complications" OR "complication") ("Kidney Transplantation"[MeSH Terms] OR "Kidney Transplantation"[All Fields] OR "Renal Transplantation"[All Fields] OR "Kidney Transplantations"[All Fields] OR "Kidney Grafting"[All Fields]) AND ("new onset diabetes"[All Fields] OR "posttransplant diabetes mellitus"[All Fields] OR "posttransplant diabetes"[All Fields] OR "post-transplant diabetes mellitus"[All Fields] OR "post-transplant diabetes"[All Fields]) AND ("Prognosis"[MeSH Terms] OR ("Prognosis"[MeSH Terms] OR "Prognosis"[All Fields] OR "prognoses"[All Fields]) OR ("Prognosis"[MeSH Terms] OR "Prognosis"[All Fields] OR "prognoses"[All Fields]) OR "prognostic factor"[All Fields] OR "Mortality"[MeSH Terms] OR ("Mortality"[MeSH Terms] OR "Mortality"[All Fields] OR "mortalities"[All Fields] OR "Mortality"[MeSH Subheading]) OR ("Mortality"[MeSH Terms] OR "Mortality"[All Fields] OR "mortalities"[All Fields] OR "Mortality"[MeSH Subheading]) OR "Survival"[MeSH Terms] OR ("Mortality"[MeSH Subheading] OR "Mortality"[All Fields] OR "Survival"[All Fields] OR "Survival"[MeSH Terms] OR "survivability"[All Fields] OR "survivable"[All Fields] OR "survivals"[All Fields] OR "survive"[All Fields] OR "survived"[All Fields] OR "survives"[All Fields] OR "surviving"[All Fields]) OR "Graft Survival"[MeSH Terms] OR "Graft Survival"[All Fields] OR "graft survivals"[All Fields] OR "complications"[All Fields] OR "complication"[All Fields])  3 "Prognosis"[Mesh] OR prognoses OR prognosis OR "prognostic factor" OR "Mortality"[Mesh] OR mortality OR mortalities OR "Survival"[Mesh] OR survival OR "Graft Survival"[Mesh] OR "graft survival" OR "graft survivals" OR "complications" OR "complication" "Prognosis"[MeSH Terms] OR "Prognosis"[MeSH Terms] OR "Prognosis"[All Fields] OR "prognoses"[All Fields] OR "Prognosis"[MeSH Terms] OR "Prognosis"[All Fields] OR "prognoses"[All Fields] OR "prognostic factor"[All Fields] OR "Mortality"[MeSH Terms] OR "Mortality"[MeSH Terms] OR "Mortality"[All Fields] OR "mortalities"[All Fields] OR "Mortality"[MeSH Subheading] OR "Mortality"[MeSH Terms] OR "Mortality"[All Fields] OR "mortalities"[All Fields] OR "Mortality"[MeSH Subheading] OR "Survival"[MeSH Terms] OR "Mortality"[MeSH Subheading] OR "Mortality"[All Fields] OR "Survival"[All Fields] OR "Survival"[MeSH Terms] OR "survivability"[All Fields] OR "survivable"[All Fields] OR "survivals"[All Fields] OR "survive"[All Fields] OR "survived"[All Fields] OR "survives"[All Fields] OR "surviving"[All Fields] OR "Graft Survival"[MeSH Terms] OR "Graft Survival"[All Fields] OR "graft survivals"[All Fields] OR "complications"[All Fields] OR "complication"[All Fields]  2 "new onset diabetes" OR "posttransplant diabetes mellitus" OR "posttransplant diabetes" OR "post-transplant diabetes mellitus" OR "post-transplant diabetes" "new onset diabetes"[All Fields] OR "posttransplant diabetes mellitus"[All Fields] OR "posttransplant diabetes"[All Fields] OR "post-transplant diabetes mellitus"[All Fields] OR "post-transplant diabetes"[All Fields]  1 "Kidney Transplantation"[Mesh] OR "Kidney Transplantation" OR "Renal Transplantation" OR "Kidney Transplantations" OR "Kidney Grafting" "Kidney Transplantation"[MeSH Terms] OR "Kidney Transplantation"[All Fields] OR "Renal Transplantation"[All Fields] OR "Kidney Transplantations"[All Fields] OR "Kidney Grafting"[All Fields] |
| **Cochrane Library (n=194)** |
| #1 "Kidney Transplantation" OR "Renal Transplantation" OR "Kidney Transplantations" OR "Kidney Grafting"  #2 "new onset diabetes" OR "posttransplant diabetes mellitus" OR "posttransplant diabetes" OR "post-transplant diabetes mellitus" OR "post-transplant diabetes"  #3 prognoses OR prognosis OR "prognostic factor" OR mortality OR mortalities OR survival OR "graft survival" OR "graft survivals" OR "complications" OR "complication"  #4 #1 AND #2 AND #3 |
| **Scopus (n=1058)** |
| TITLE-ABS-KEY ( "Kidney Transplantation" OR "Renal Transplantation" OR "Kidney Transplantations" OR "Kidney Grafting" ) AND TITLE-ABS-KEY ( "new onset diabetes" OR "posttransplant diabetes mellitus" OR "posttransplant diabetes" OR "post-transplant diabetes mellitus" OR "post-transplant diabetes" ) AND TITLE-ABS-KEY ( "prognoses" OR "prognosis" OR "prognostic factor" OR "mortality" OR "mortalities" OR "survival" OR "graft survival" OR "graft survivals" OR "complications" OR "complication" ) |
| **Web of Science (n=787)** |
| 1: TS=("Kidney Transplantation" OR "Renal Transplantation" OR "Kidney Transplantations" OR "Kidney Grafting")  2: TS=("new onset diabetes" OR "posttransplant diabetes mellitus" OR "posttransplant diabetes" OR "post-transplant diabetes mellitus" OR "post-transplant diabetes")  3: TS=(prognoses OR prognosis OR "prognostic factor" OR mortality OR mortalities OR survival OR "graft survival" OR "graft survivals" OR "complications" OR "complication")  4: #3 AND #2 AND #1 |
| **Ovid-MEDLINE (n=871)** |
