# Supplementary material for: Prognostic impact of post-transplant diabetes mellitus in kidney allograft recipients: a meta-analysis
Source: Nephrol Dial Transplant. 2024 Aug 12;40(3):554–76. doi: 10.1093/ndt/gfae185 (PMC11879034; doi:10.1093/ndt/gfae185)
Supplement: gfae185_Supplemental_Files [file gfae185_supplemental_files.zip › Supplementary Table 3. NOS assessment of the included studies.docx]

**Supplementary Table S2: Quality assessment of studies using the Newcastle-Ottawa Scale for cohort and case-control studies**

| **Study** | **Study**  **Design** | **Selection** | | | | **Comparability** | **Outcome** | | | **Total Score** |
| --- | --- | --- | --- | --- | --- | --- | --- | --- | --- | --- |
|  |  | *Representative of exposed cohort* | *Selection of non-exposed cohort* | *Ascertainment of exposure* | *Outcome not present at start* | *On the basis of design or analysis* | *Assessment of outcome* | *Follow-up >1 year* | *Follow-up adequate* |  |
| Abu Elmagd, 2008 | Case-control | * |  | * | * | * | * | * | n/a | **6** |
| Cotovio, 2013 | Case-control | * | * | * | * | * | * | * | n/a | **7** |
| Einollahi, 2008 | Case-control | * | * |  | * | ** | * | * | n/a | **7** |
| Nagib, 2015 | Case-control | * |  | * | * | * | * | * | n/a | **6** |
| Romagnoli, 2005 | Case-control | * | * |  | * |  | * | * | n/a | **5** |
| Sharma, 2003 | Case-control | * | * |  | * |  | * | * | n/a | **5** |
| Siraj, 2010 | Case-control | * | * |  | * |  | * | * | n/a | **5** |

| **Study** | **Study**  **Design** | **Selection** | | | | **Comparability** | **Outcome** | | | **Total Score** |
| --- | --- | --- | --- | --- | --- | --- | --- | --- | --- | --- |
|  |  | *Representative of exposed cohort* | *Selection of non-exposed cohort* | *Ascertainment of exposure* | *Outcome not present at start* | *On the basis of design or analysis* | *Assessment of outcome* | *Follow-up >1 year* | *Follow-up adequate* |  |
| Ahmed, 2017 | Prospective Cohort |  | * | * | * |  | * |  | * | **5** |
| Al-Ghareeb, 2012 | Retrospective cohort | * | * | * | * |  | * | * | * | **7** |
| Alagbe, 2017 | Retrospective cohort | * | * | * | * |  | * | * | * | **7** |
| Baron, 2017 | Retrospective cohort | * | * | * | * |  | * | * | * | **7** |
| Bzoma, 2018 | Retrospective cohort | * | * | * | * | * | * |  | * | **7** |
| Cheng, 2020 | Retrospective cohort | * | * | * | * |  | * | * | * | **7** |
| Cheng, 2022 | Prospective Cohort | * | * | * | * |  | * | * | * | **7** |
| Choi, 2013 | Retrospective cohort | * | * | * | * | * | * | * | * | **8** |
| Cosio, 2002 | Retrospective cohort | * | * | * | * | ** | * | * | * | **9** |
| Dedinská, 2015 | Retrospective cohort |  | * | * | * | * | * | * | * | **7** |
| Demirci, 2010 | Retrospective cohort | * | * | * | * | * | * | * | * | **8** |
| Dienemann, 2016 | Retrospective cohort | * | * | * | * | * | * | * | * | **8** |
| Ducloux, 2005 | Prospective cohort | * | * | * | * | ** | * | * | * | **9** |
| Fernández-Fresnedo, 2003 | Retrospective cohort | * | * |  | * |  |  | * | * | **5** |
| Gnatta, 2010 | Prospective cohort | * | * | * | * |  | * |  | * | **6** |
| Gonzalez-Posada, 2004 | Retrospective cohort | * | * | * | * | ** | * | * | * | **9** |
| González-Posada, 2006 | Retrospective cohort | * | * | * | * | ** | * | * | * | **9** |
| Hussain, 2022 | Retrospective cohort | * | * | * | * | ** | * | * |  | **8** |
| Jeon, 2023 | Retrospective cohort | * | * | * | * | ** | * | * | * | **9** |
| John, 2001 | Prospective cohort | * | * | * | * | * | * | * | * | **8** |
| Johny, 2002 | Retrospective cohort | * | * | * | * |  | * | * | * | **7** |
| Joss, 2007 | Retrospective cohort | * | * | * | * |  | * | * | * | **7** |
| Kasiske, 2003 | Retrospective cohort |  | * | * | * | ** | * | * | * | **8** |
| Khalkhali, 2010 | Retrospective cohort | * | * | * | * | ** | * | * | * | **9** |
| Kumar, 2020 | Prospective cohort |  | * | * | * | * | * | * | * | **7** |
| Lim, 2021 | Retrospective cohort | * | * | * | * | ** | * | * | * | **9** |
| Lv, 2014 | Retrospective cohort | * | * | * | * | ** | * | * | * | **9** |
| Maekawa, 2020 | Retrospective cohort |  | * | * | * | ** | * | * | * | **8** |
| Malik, 2021 | Prospective cohort |  | * | * | * | * | * | * |  | **6** |
| Miles, 1998 | Retrospective cohort | * | * | * | * | ** | * | * | * | **9** |
| Nagaraja, 2013 | Retrospective cohort |  | * | * | * | ** | * | * | * | **8** |
| Nie, 2019 | Retrospective cohort | * | * | * | * |  | * | * | * | **7** |
| Ouni, 2022 | Retrospective cohort | * | * | * | * |  | * | * | * | **7** |
| Park, 2015 | Retrospective cohort | * | * | * | * |  | * | * | * | **7** |
| Porrini, 2019 | Retrospective cohort | * | * | * | * | ** | * | * | * | **9** |
| Rosettenstein, 2016 | Prospective cohort | * | * | * | * |  | * |  | * | **6** |
| Roth, 1998 | Retrospective cohort | * | * | * | * | * | * | * |  | **7** |
| Savaj, 2008 | Retrospective cohort | * | * | * | * |  | * | * | * | **7** |
| Sezer, 2006 | Retrospective cohort | * | * | * | * | * | * | * | * | **8** |
| Sheu, 2016 | Retrospective cohort | * | * | * | * |  | * | * | * | **7** |
| Silva, 2000 | Retrospective cohort | * | * | * | * |  | * | * | * | **7** |
| Sulanc, 2005 | Retrospective cohort | * | * | * | * |  | * | * | * | **7** |
| Tsai, 2011 | Retrospective cohort | * | * | * | * |  | * | * | * | **7** |
| Tutone, 2004 | Retrospective cohort | * | * | * | * | ** | * | * | * | **9** |
| Veroux, 2013 | Retrospective cohort | * | * | * | * |  | * | * | * | **7** |
| Wauters, 2012 | Retrospective cohort | * | * | * | * | ** | * | * | * | **9** |
| Yeh, 2020 | Retrospective cohort | * | * | * | * | ** | * |  |  | **7** |
| Demir, 2022** | Retrospective cohort |  | * | * | * |  |  |  |  | **3** |

*Total scores indicate study quality: 1-4 (poor), 5-6 (fair), and 7-9 (good).*

***This study graded as poor and therefore cannot be included in the meta-analysis.*
