# Supplementary figures and images for: Prognostic impact of post-transplant diabetes mellitus in kidney allograft recipients: a meta-analysis
Source: Nephrol Dial Transplant. 2024 Aug 12;40(3):554–76. doi: 10.1093/ndt/gfae185 (PMC11879034; doi:10.1093/ndt/gfae185)

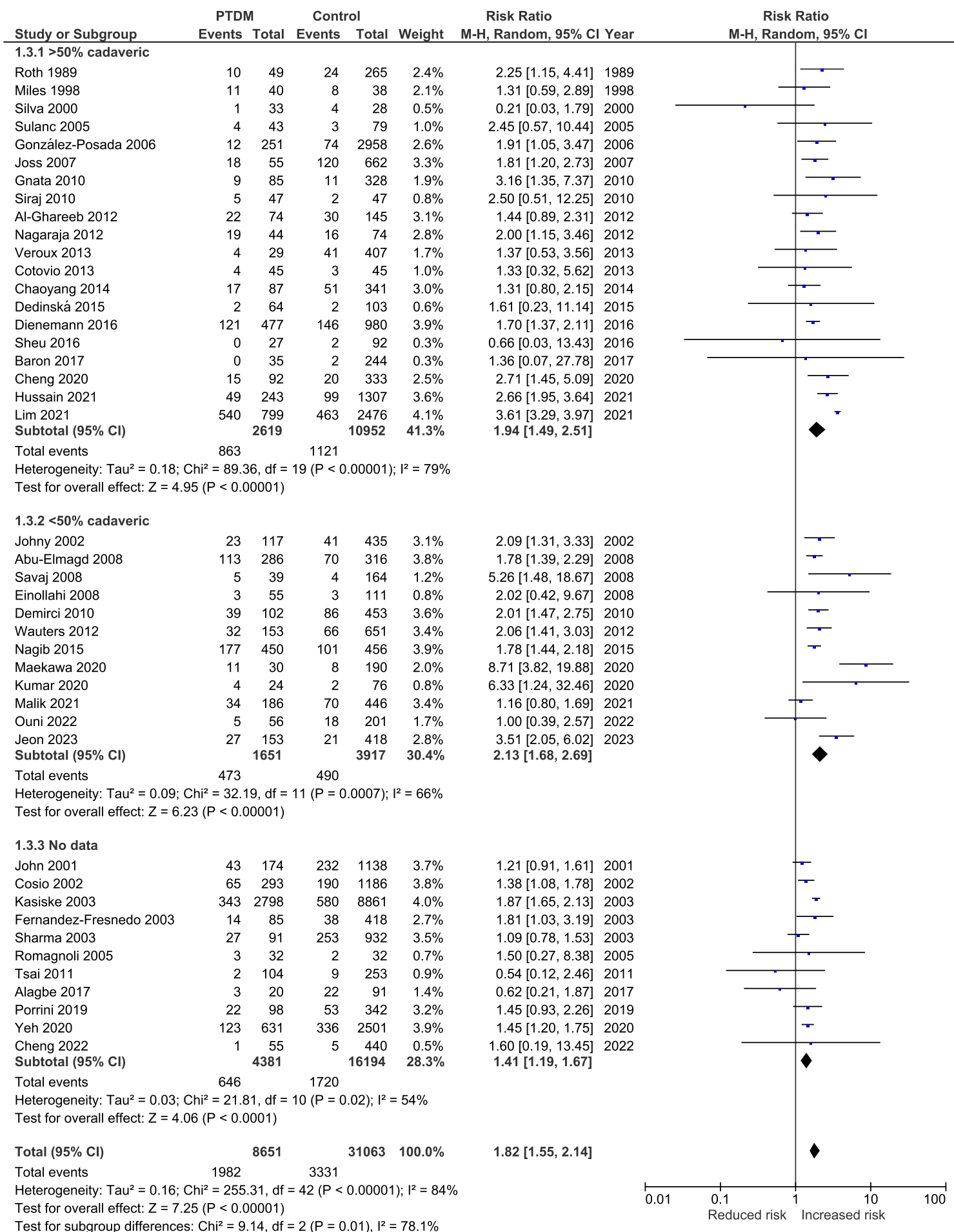

Supplement: gfae185_Supplemental_Files [file gfae185_supplemental_files.zip › Supplementary Figure 1 All-cause mortality - supplementary - New.pdf]

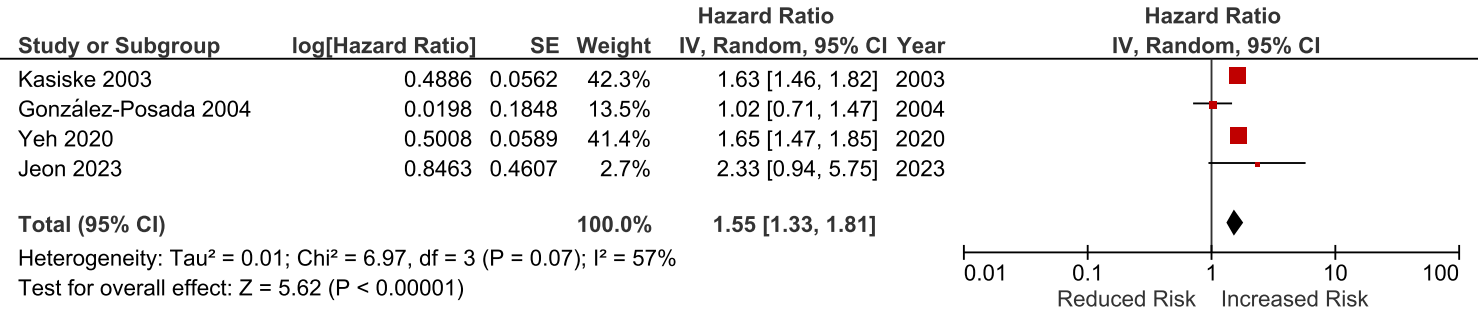

Supplement: gfae185_Supplemental_Files [file gfae185_supplemental_files.zip › Supplemtary Figure 2 Graft loss - supplementary.pdf]
